# Three new species of EntolomasubgenusPouzarella from China based on morphological and molecular data

**DOI:** 10.3897/mycokeys.44.24998

**Published:** 2018-12-13

**Authors:** Xiao-Lan He, Egon Horak, Di Wang, Wei-Hong Peng, Bing-Cheng Gan

**Affiliations:** 1 Soil and Fertilizer Institute, Sichuan Academy of Agricultural Sciences, Chengdu, 610066, China Soil and Fertilizer Institute, Sichuan Academy of Agricultural Sciences Chengdu China; 2 Schlossfeld 17 A-6020 Innsbruck, Austria Unaffiliated Innsbruck Austria

**Keywords:** Entolomataceae, systematics, taxonomy, multi-gene analyses

## Abstract

In the present paper, three additional species of EntolomasubgenusPouzarella viz. *E.erectoides, E.griseocarpum* and *E.rubropilosum* are described from China. *E.rubropilosum* is a typical species in section Pouzarella; *E.griseocarpum* and *E.erectoides* are members of sect. Dysthales. The taxa are further confirmed by ITS, RPB2, LSU and mtSSU analyses and phylogenetic relationships with other Entolomasubgen.Pouzarella species are also discussed. ITS sequence analysis showed that the sizes of the entire ITS region and ITS1 are remarkably divergent, while the ITS2 is conserved in length within Entolomasubgen.Pouzarella. Molecular analyses, based on the combined dataset, demonstrated that species diversity of subgen.Pouzarella in China is much higher than previously thought, in the present study twenty phylogenetic species from China are taken into consideration. On the other hand, morphological and molecular analyses suggested that classification of Entolomasubgen.Pouzarella probably has to be fundamentally re-adjusted based on additional data.

## Introduction

*Pouzarella* Mazzer is a distinctive group of entolomatoid species that was accepted as a genus by some researchers (Horak 2008; Largent 1994; Mazzer 1976). Others consider it as a subgenus of *Entoloma* P. Kumm. (Noordeloos 1979, 1992, 2004; Noordeloos and Gates 2012). Recent molecular phylogenetic studies (Co-David et al. 2009; Baroni and Matheny 2011; He et al. 2013; Largent and Bergemann 2015) based on multi-loci showed that *Pouzarella* actually represent a distinct monophyletic group separated from the other entolomatoid groups. In addition, taxa of *Pouzarella* are easily recognised both by macro- and micromorphological characters. However, here we still treat it as a subgneus of *Entoloma* s.l. because accepting *Pouzarella* as a separate genus would make *Entoloma* s.l. paraphyletic. Taxonomical revision of other well supported, distinct clades of *Entoloma* s.l. is therefore needed before a formal decision on the generic status of *Pouzarella* can be made. When such a revision is achieved, we predict, that *Pouzarella* will be a well-defined genus based on morphological and molecular evidence. In the field, species of Entolomasubgen.Pouzarella are often overlooked due to their small and dull-coloured basidiomes. However, the inconspicuous species are widespread and have been reported from tropical to temperate regions. So far, more than seventy species have been described worldwide (Mazzer 1976; Horak 1980, 1983, 2008; Desjardin and Baroni 1991; Noordeloos 1992, 2004; Noordeloos et al. 1992; Manimohan et al. 1995; Baroni and Ortiz 2002; Manimohan et al. 2006; Karstedt et al. 2007; Baroni et al. 2008; Largent et al. 2011; Baroni et al. 2012; Noordeloos and Gates 2012; He et al. 2013; Largent and Bergemann 2015; Raj and Manimohan 2017).

Basidiomes of members in subgen.Pouzarella are easy to recognise. However, many species have in common small basidiome size and greyish colours and, therefore, it is difficult to distinguish them to species by morphological characters only. Accordingly, both morphological and molecular data are needed to refine the species concept and understand the diversity of these small agarics in Entolomasubgen.Pouzarella.

In previous studies, seven species of Entolomasubgen.Pouzarella were reported to occur in China (Ying 1995; He et al. 2013). However, we believe that subgen.Pouzarella remains poorly understood in this region, rich in many diverse ecological habitats. In continuation of previous surveys, further field work was carried out in southwest and northeast China. More than 50 samples matching the concept of Entolomasubgen.Pouzarella were collected and many turned out to be different from the locally already known species. As a first step, three distinctive new taxa are described in the present study whereas the other specimens were shelved for the moment because of scarcity of material. To further confirm the three new taxa and infer the affinities amongst representative species of Entolomasubgen.Pouzarella, phylogenetic analysis was carried out based on the combination of ITS, RPB2, mtSSU and nLSU sequences.

## Materials and methods

### Morphological descriptions

Fresh basidiomes were photographed in the field and described macroscopically. Colour notations follow Kornerup and Wanscher (1978). Microscopic examination was done using a Leica DM5000B microscope. Basidiospores, basidia and pileipellis were mounted and measured in 5% potassium hydroxide (KOH) and/or 1% Congo Red. Pigmentation of the micro-structures was observed in distilled water. Measurements of the basidiospores excluded hilar appendix (apiculus) and at least 30 basidiospores of each specimen were measured. Q represents the length to width ratio of a basidiospore in profile view; ***Q*** represents the average Q of all basidiospores and is given ± standard deviation; *x* represents the means of basidiospore length and width ± standard deviation. All cited collections, including the holotypes, are deposited at the Mycological Herbarium of Soil and Fertilizer Institute, Sichuan Academy of Agricultural Sciences (SAAS), Chengdu, China and the Herbarium ZT of ETH Zurich, Switzerland.

### DNA extraction, PCR amplification and sequencing

Genomic DNA was extracted with Biospin Fungus Genomic DNA Extraction Kit following the manufacturer’s instructions. PCR amplification was performed using DreamTaq™ Green PCR Master qMix (2×), Fermentas. The primers for RPB2 amplification were rpb2-6F and rpb2-7R, rpb2-i6f and rpb2-i7r (Matheny 2005; Co-David et al. 2009). ITS regions were amplified with ITS5 and ITS4; LR0R and LR5, MS1 and MS2 were used for nLSU and mtSSU amplification, respectively (White et al. 1990; http://www.biology.duke.edu/fungi/mycolab/primers.htm).

### Sequence alignment and phylogenetic analyses

Sequences used in phylogenetic analyses are listed in Table 1 and aligned in muscle 3.6 (Edgar 2004). The aligned sequences were manually modified where necessary in Mega 6.0 (Tamura et al. 2013). Phylogenetic analyses were based on the combined ITS, nLSU, RPB2 and mtSSU sequences. For the ITS region, only ITS1 and ITS2 were kept for further analyses. Conflicts between the ITS, nLSU, RPB2 and mtSSU datasets were evaluated by comparing the topologies resulting from the phylogenetic analysis of the single gene. As no conflict was detected amongst the well supported clades of the different trees, sequences of the four genes were combined for further analyses.

*Maximum likelihood analysis* – ML analysis was carried out by the web RAxML Version 8 (http://www.phylo.org/sub_sections/portal/) under the GTRGAMMAI model with 1000 bootstrap replicates (Stamatakis 2014). “Find best tree using maximum likelihood search” option was selected when analysis was undertaken.

*Maximum parsimony analysis* – MP analysis was performed using PAUP* version 4.0b10 (Swofford 2003). All characters were treated as unordered and of equal weight. Gaps were treated as missing data. Bootstrap values (BS) were obtained from 1000 replicates.

*Bayesian analysis* – Bayesian analysis was performed using MrBayes 3.2.6 (Ronquist and Huelsenbeck 2003). The best substitution models for each marker were selected using the Akaike Information Criterion (AIC) in jModelTest 2.1.7 (Darriba et al. 2012). GTR+I+G model was selected for nLSU, GTR+G for mtSSU and ITS and SYM+G for RPB2. Two runs of six Markov chains were run from random starting trees for 6 million generations and sampled every 100 generations. Every time the diagnostics were calculated, 25% of the samples from the beginning of the chain were discarded. Runs were stopped after the average standard deviation was below 0.01. Bayesian posterior probabilities (BPP) were determined after calculating a 75% majority rule consensus tree.

**Table 1.** A list of taxa, specimens and GenBank accession numbers of sequences used in this study.

Taxa	Collection No.	Origin	GenBank accessions				Remarks
			ITS	LSU	RPB2	mtSSU	
* Entoloma albostrigosum *	DL Largent 9641	Australia: Queensland	–	HQ876535	HQ876513	HQ876557	GenBank ID: *Pouzarellaalbostrigosa*
	DL Largent 9663	Australia: Queensland	–	HQ876536	HQ876514	HQ876558	GenBank ID: *P.albostrigosa*
* E. araneosum *	ME Noordeloos 200314	China: Jilin	KC710056	GQ289153	GQ289225	GQ289293	
* E. barringtonense *	DL Largent 9901 (Holotype)	Australia: Queensland	–	HQ876524	HQ876543	HQ876546	GenBank ID: *P.parvula*
* E. changchunense *	HMJAU 3886 (Holotype)	China: Jilin	–	JQ993095	–	JQ993061	
* E. crassicystidiatum *	GDGM 28821	China: Guangdong	KC678997	JQ291567	JQ993085	JQ993058	
	GDGM 27357 (Holotype)	China: Guangdong	KC678996	JQ291569	JQ993083	JQ993056	
* E. debile *	DLL 9784	Australia: Queensland	–	HQ876528	HQ876506	HQ876550	GenBank ID: *P.debilis*
* E. dindenense *	DL Largent 9623	Australia: Queensland	–	HQ876527	HQ876505	HQ876549	GenBank ID: *P.fusca*
* E. erectoides *	SAAS 1232 (Holotype)	China: Sichuan	**MH020746**	**KU534255**	**KU534496**	–	
	SAAS 945	China: Sichuan	**MH020769**	**KU534239**	**KU534498**	–	
	SAAS 1361	China: Jilin	**MH020755**	–	**KU534484**	–	
* E. farinosum *	DL Largent 9934 (Holotype)	Australia: Queensland	–	HQ876516	HQ876495	HQ876538	GenBank ID: *P.farinosa*
	DL Largent 9900	Australia: Queensland	–	HQ876515	HQ876494	HQ876537	GenBank ID: *P.farinosa*
* E. furfuraceum *	GDGM 28818 (Holotype)	China: Jinlin	JX975293	JQ993094	JQ993084	JQ993062	
	SAAS 104	China: Jilin	–	KU534240	–	–	
* E. griseocarpum *	SAAS 1230	China: Tibet	**MH020753**	**KU534253**	**KU534500**	**KU534438**	
	SAAS 1328 (Holotype)	China: Tibet	**MH020766**	**KU534256**	**KU534501**	**KU534455**	
	SAAS 951	China: Sichuan	**MH020770**	**KU534242**	**KU534499**	**KU534457**	
* E. lageniforme *	DL Largent 9895	Australia: Queensland	–	HQ876523	HQ876502	HQ876545	GenBank ID: *P.lageniformis*
* E. lasium *	DL Largent 9662	Australia: Queensland	–	HQ876529	HQ876507	HQ876551	GenBank ID: *P.lasia*
	DL Largent 9670	Australia: Queensland	–	HQ876530	HQ876508	HQ876552	GenBank ID: *P.lasia*
	DL Largent 9807	Australia: Queensland	–	HQ876533	HQ876511	HQ876555	GenBank ID: *P.lasia*
	DL Largent 9811	Australia: Queensland	–	HQ876534	HQ876512	HQ876556	GenBank ID: *P.lasia*
	DL Largent 9729	Australia: Queensland	–	HQ876531	HQ876509	HQ876553	GenBank ID: *P.lasia*
	DL Largent 9778	Australia: Queensland	–	HQ876532	HQ876510	HQ876554	GenBank ID: *P.lasia*
* E. nodosporum *	TENN:068582	USA: Tennessee	KY744163	MF797654	–	–	GenBank ID: *P.nodospora*
* E. pamiae *	DL Largent 9794	Australia: Queensland	–	HQ876517	HQ876496	HQ876539	GenBank ID: *P.pamiae*
	DL Largent 9834	Australia: Queensland	–	HQ876519	HQ876498	HQ876541	GenBank ID: *P.pamiae*
	DL Largent 9808	Australia: Queensland	–	HQ876518	HQ876497	HQ876540	GenBank ID: *P.pamiae*
* E. perbloxamii *	MEN 2004071 (Holotype)	Australia: Tasmania	KC710117	GQ289178	GQ289249	GQ289318	
* E. pilocystidiatum *	DL Largent 9848	Australia: Queensland	–	HQ876520	HQ876499	HQ876542	GenBank ID: *P.pilocystidiata*
* E. pilocystidiatum *	DL Largent 9932 (Holotype)	Australia: Queensland	–	HQ876521	HQ876500	HQ876543	GenBank ID: *P.pilocystidiata*
	DL Largent 9949	Australia: Queensland	–	HQ876522	HQ876501	HQ876544	GenBank ID: *P.pilocystidiata*
* E. prunuloides *	MEN 200340	Slovakia	KC710073	GQ289184	GQ289255	GQ289324	
* E. rubropilosum *	SAAS 406 (Holotype)	China: Sichuan	**MH020761**	**KU534218**	**KU534488**	**KU534439**	
	SAAS 1112	China: Tibet	**MH020767**	**KU534252**	**KU534502**	**KU534454**	
* E. setiforme *	DL Largent 9809 (Holotype)	Australia: Queensland	–	HQ876525	HQ876503	HQ876547	GenBank ID: *P.setiformis*
	DL Largent 9810	Australia: Queensland	–	HQ876526	HQ876504	HQ876548	GenBank ID: *P.setiformis*
* E. silvanum *	K(M) 191739 (Holotype)	India: Kerala	KY643747	KY643724	–	–	
*E.* sp. 1	SAAS 894	China: Sichuan	**MH020765**	**KU534245**	**KU534491**	**KU534447**	
*E.* sp. 2	SAAS 1088	China: Jilin	**MH020749**	**KU534246**	–	**KU534441**	
	SAAS 1210	China: Jilin	**MH020752**	**KU534248**	–	**KU534449**	
*E.* sp. 3	SAAS 249	China: Sichuan	**MH020759**	**KU534243**	–	–	
*E.* sp. 4	SAAS 1209	China: Jilin	**MH020751**	–	**KU534492**	**KU534448**	
	SAAS 291	China: Jilin	**MH020760**	–	**KU534486**	**KU534444**	
*E.* sp. 5	SAAS 1360	China: Jilin	**MH020754**	**KU534249**	–	**KU534456**	
*E.* sp. 6	SAAS 1464	China: Sichuan	**MH020756**	**KU534258**	**KU534493**	**KU534450**	
*E.* sp. 7	SAAS 100	China: Sichuan	**MH020747**	–	–	**KU534442**	
*E.* sp. 8	080301	China: Sichuan	**MH020745**	**KU534254**	–	–	
	SAAS 102	China: Sichuan	**MH020748**	–	**KU534489**	**KU534443**	
*E.* sp. 9	SAAS 1527	China: Shaanxi	**MH020758**	**KU534251**	**KU534495**	**KU534452**	
*E.* sp. 10	SAAS 529	China: Sichuan	**MH020763**	**KU534244**	**KU534497**	**KU534446**	
	SAAS 772	China: Sichuan	**MH020764**	**KU534241**	**KU534487**	**KU534458**	
*E.* sp. 11	SAAS 526	China: Shaanxi	**MH020762**	**KU534257**	**KU534490**	**KU534445**	
* E. strigosissimum *	152	Italy	JF908004	–	–	–	
* E. subaraneosum *	GDGM 28823 (Holotype)	China: Jilin	JQ320113	JQ410329	–	–	
	KA 12-1534	South Korea	KJ523135	–	–	–	
* E. tenuissimum *	GDGM 28813	China: Jilin	JX975295	JQ993097	JQ993086	JQ993059	
	GDGM 28814 (Holotype)	China: Heilongjiang	JX975294	JQ993096	JQ993087	JQ993060	
* E. violaceovillosum *	P. Manomohan 645 (Holotype)	India: Kerala	–	GQ289205	GQ289273	GQ289345	
* E. yunnanense *	GDGM 28815	China: Yunnan	JQ320108	JQ320128	–	JQ993057	

Sequences in bold and marked with “KU” and “MH” are newly generated in this study. Sequences marked with “GQ” were from Co-David et al. (2009). Sequences marked with “HQ” were from Largent et al. (2011). KC710056, KC710073 and KC710117 were from Morgado et al. (2013). KC678996, KC678997 and sequences marked with “JQ” and “JX” were from He et al. (2013). KY643747 and KY643724 were from Raj and Manimohan (2017). JF908004 was from Osmundson et al. (2013). KJ523135 was from Kim et al. (2015). KY744163 and MF797654 are unpublished.

## Taxonomy

### Entoloma erectoides

Taxon classificationFungiAgaricalesEntolomataceae

Xiao L. He & E. Horak sp. nov.

828692

Figs 1a, b , 2

#### Diagnosis.

*E.erectoides* is distinguished by the greyish brown pileus covered with silvery fibrils, large basidiospores (13.5–17.5 × 8–9.5 µm) and presence of ovoid to subutriform cheilocystidia.

#### Type.

CHINA. SICHUAN PROV.: Yajiang County, Gexigou National Nature Reserve, 29°33'N, 100°50'E, elevation ca. 2980 m, August 2014, He XL (SAAS 1232, holotype; ZT 14180, isotype).

#### Etymology.

*Erectoides*, refers to the suberect to erect fibrils on the pileus.

**Figure 1.**
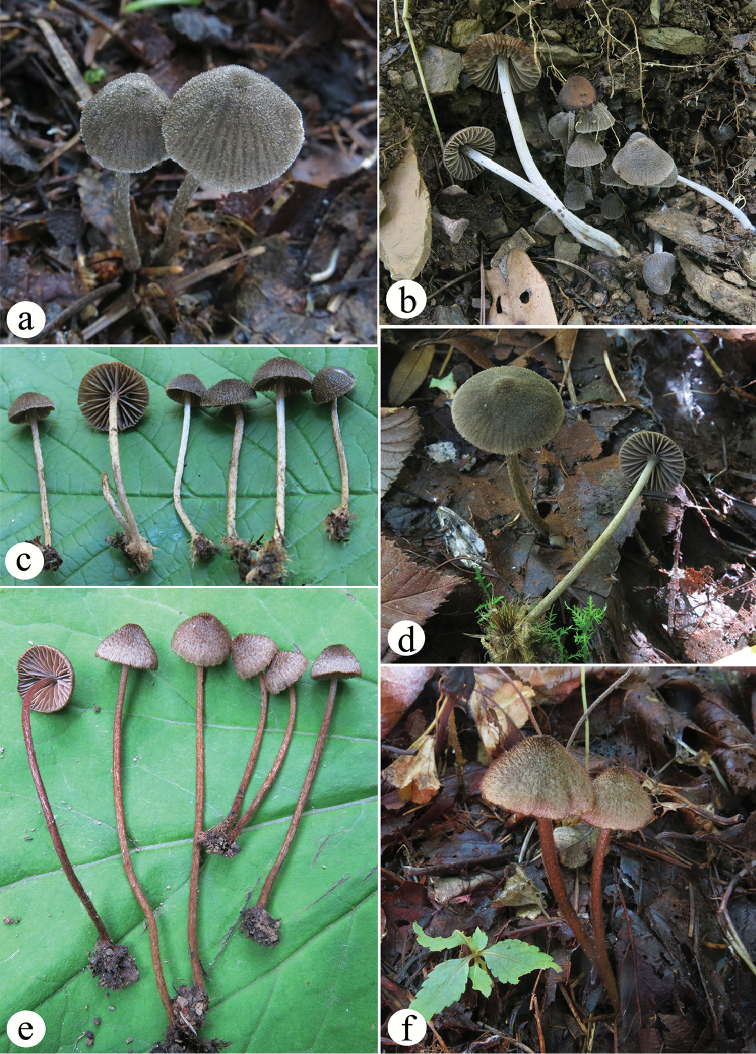
Basidiomata of the new species. **a, b***E.erectoides* (SAAS 1361, SAAS 1232) **c, d***E.griseocarpum* (SAAS 951, SAAS 1328) **e, f***E.rubropilosum* (SAAS 406, SAAS 1112).

#### Description.

Pileus 5–15 mm broad, bluntly conic, convex or campanulate, dry, slightly hygrophanous, greyish-brown to brown (5C2–5D2), densely covered with suberect fibrils or minutely fibrillose squamules; fibrils silvery greyish, striate from entire margin to near centre. Lamellae sinuate, ventricose, distant, up to 3.5 mm wide, moderately thick, with two tiers of lamellulae, dark grey to brownish-grey, with entire and concolorous edge. Stipe 40–60 × 1–2.5 mm, central, cylindrical, equal, dry, concolorous with pileus, densely covered with grey to greyish fibrils, hollow, surface dry, with a pale yellow brownish to pale brownish strigose base. Context thin, concolorous with pileus. Odour and taste not distinctive.

Basidiospores (13–) 13.5–17.5 × 8–9.5 (–10.5) µm (*x* = 15.5 ± 0.5 × 8.8 ± 0.3 µm), Q = 1.50–1.94 (***Q*** = 1.72 ± 0.03), heterodiametric, strongly angled in profile and face views with 6–9 facets, appearing nodulose, pale yellow brownish, thick-walled. Basidia 39–48 × 13–18 µm, subclavate or clavate, 4-spored. Aborted basidia scattered in the hymenium, often filled with dark brown amorphous cytoplasmic pigment. Lamellar trama dark brown, composed of parallel, cylindrical, heavily encrusted and thin-walled cells, 6–15 µm wide. Lamellar edge sterile. Cheilocystidia 33–90 × 12–33 µm, broadly ovoid to utriform (32–65 × 22–30 µm) or lageniform (70–90 × 16–20 µm), with pale brownish, intracellular pigment, slightly thick-walled. Pileipellis a trichoderm composed of clustered and suberect hyphae, walls externally encrusted with brown pigment; terminal cells 30–50 (–90) × 8–15 µm, cylindrical to slightly fusoid; subpellis composed of cylindrical, encrusted hyphae, up to 20 µm broad. Stipitipellis composed of thin-walled and pale yellowish-brown encrusted hyphae; terminal cells 40–80 × 9–15 µm, cylindrical to slender fusoid, walls encrusted with pale yellow-brown pigment. Oleiferous hyphae absent. Clamp connections absent.

#### Habitat.

Scattered or gregarious on soil and amongst leaf litter in broadleaf forest dominated by *Quercus* or on soil amongst decaying leaves of *Betula*, *Pandus* and *Abies*.

#### Additional collections examined.

CHINA. SICHUAN PROVINCE: CHINA: SICHUAN PROV. Yajiang County, Gexigou National Nature Reserve, 29°33'N, 100°50'E, elevation ca. 2980 m, 24 July 2013, He X.L. (SAAS 945). JILIN PROV.: Antu County, Changbai Mountains, 42°10'N, 127°55'E, elevation ca. 750 m, 25 August 2014, He X.L. (SAAS 1361).

#### Comments.

Morphologically, *Entolomaerectoides* is a member of section Dysthales. In literature, a few species in section Dysthales are described having silvery fibrils or squamules on pileus and stipe. Accordingly, *E.erectoides* can be confused with the Argentinean *E.calileguense* Blanco-Dios (as *Pouzarellavariabilis* T.J. Baroni, Albertó, Niveiro & B.E. Lechner in Baroni et al. 2012). Both species have silvery greyish-brown erect fibrils or squamules on the pileus and stipe. However, the latter species is easily separated by the much larger basidiospores (16–23.5 × 10–12 μm, Baroni et al. 2012). *E.farinosum* (Largent & Skye Moore) Noordel. & G.M. Gates, reported from Australia, differs by globose or nearly napiform cheilocystidia (Largent et al. 2011). In addition, this taxon is separated from *E.erectoides* by molecular evidence. *E.tenuissimum* T.H. Li & Xiao-Lan He, also recorded from China, is distinguished by the smaller and slimmer basidiomes and taxonomically is also distinctly different based on molecular analysis (He et al. 2013). *E.argenteolanatum* (T.J. Baroni, Perd.-Sánch. & S.A. Cantrell) Noordel. & Co-David was found on decaying leaves of tropical trees and shrubs in the Dominican Republic and is characterised by denser and longer silvery fibrils and the place of discovery in the Caribbean on the island of Hispaniola (Baroni et al. 2008). The other grey-brown species with silvery fibrils in section Versatile could be distinguished by the innately fibrillose pileus and stipe and colourless hymenial cystidia.

**Figure 2.**
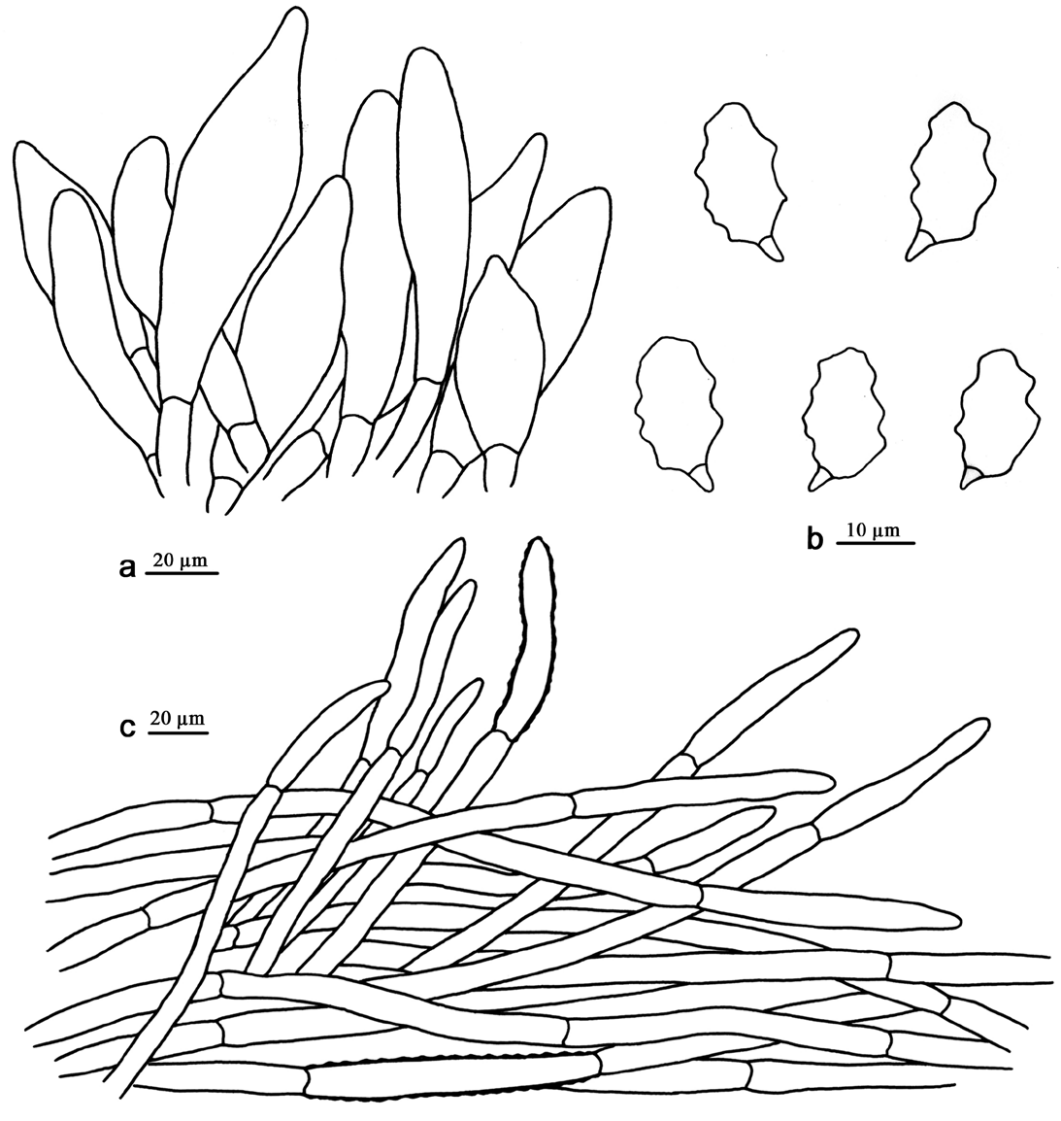
Microscopic structures of *E.erectoides* (drawn from the holotype). **a** Cheilocystidia **b** Basidiospores **c** Pileipellis.

### Entoloma griseocarpum

Taxon classificationFungiAgaricalesEntolomataceae

Xiao L. He & E. Horak sp. nov.

828701

Figs 1c, d , 3

#### Diagnosis.

*E.griseocarpum* is characterised by the greyish-brown pileus, large basidiospores (12.5–15.5 × 7.5–9 µm) and broadly clavate, ovoid to lageniform cheilocystidia.

#### Type.

CHINA. TIBET: Linzhi, Lulang, 29°94'N, 94°79'E, elevation ca. 3800 m, 18 September 2014, He X.L. (SAAS 1328, holotype).

#### Etymology.

*griseocarpum*, refers to the greyish-brown coloured basidiomes.

#### Description.

Pileus 5–20 mm broad, hemispherical, convex, bluntly conic to broadly campanulate, dry, not hygrophanous, greyish-brown to brown (4D3–4E3), densely covered by suberect hispid or minutely squamulose overall, denser in centre; fibrils dark grey, pale grey brownish or concolorous with pileal surface (4D2–4D3), striate from entire margin to near centre. Lamellae sinuate with short decurrent tooth, ventricose, distant, moderately thick, up to 3 mm broad, with two tiers of lamellulae, dark grey to brownish-grey, with entire and concolorous edges. Stipe 20–50 × 0.7–1.5 mm, cylindrical, equal, dry, concolorous with pileus, densely covered with pale yellow brownish flocculose hairs, hollow, with a dirty yellowish to pale yellow brownish strigose base. Context thin, concolorous with pileus. Odour and taste not distinctive.

Basidiospores 12.5–15.5 (–17) × (6.5–) 7.5–9 (–9.5) µm (*x* = 13.8 ± 0.3 × 8.3 ± 0.3 µm), Q = 1.60–1.94 (***Q*** = 1.71 ± 0.02), heterodiametric, strongly angled in profile and face view with 6–10 facets, appearing nodulose, pale yellow brownish, thick-walled. Basidia 35–55 × 11–13 (–15) µm, subclavate to clavate, 4-spored. Aborted basidia scattered in the hymenium, filled with dark brown amorphous cytoplasmic pigment. Lamellar trama dark brown, composed of parallel, cylindrical, heavily encrusted and thin-walled elements. Lamellar edges sterile. Cheilocystidia 23–50 × (10–) 12 20 µm, broadly clavate, ovoid to lageniform; with brownish, intracellular pigment, slightly thick-walled. Pileipellis a trichoderm composed of yellow brown, suberect and multiseptate hyphae, walls heavily encrusted with brown pigment; terminal cells 35–105 × 8–27 µm, cylindrical, subclavate or bullet-shaped, thin to moderately thick-walled; subpellis composed of cylindrical encrusted hyphae, up to 25 µm diam. Stipitipellis composed of yellow-brown encrusted hyphae; terminal cells 40–80 × 4–10 µm, slender cylindrical with obtuse apex, thin-walled, sparsely encrusted with pale yellowish-brown pigment. Oleiferous hyphae absent. Clamp connections absent.

#### Habitat.

Scattered on soil amongst decaying litter in mixed conifer-broadleaf forest dominated by *Quercus*, *Betula*, *Rhododendron* and *Abies*.

#### Additional collections examined.

CHINA. TIBET: Linzhi, Lulang, 29°94'N, 94°79'E, elevation ca. 3800 m, 18 September 2014, He X.L. (SAAS 1230, SAAS 1657, SAAS 1751, SAAS 1871). SICHUAN PROV.: Jiuzhaigou, 33°28'N, 103°59'E, elevation ca. 3000 m, 20 July 2013, He X.L. (SAAS 951).

#### Comments.

The greyish-brown pileus covered by suberect hispid or minutely squamulose, the brown external encrustations on pileipellis and stipitipellis and the cylindrical terminal cells of pileipellis and stipitipellis indicate *E.griseocarpum* belongs to the sect. Dysthales. It is very similar to *E.albostrigosum* (Largent & Abell-Davis) Blanco-Dios and *E.lasium* (Berk. & Broome) Noordel. & Co-David (Largent et al. 2011). However, *E.albostrigosum* is distinguished by the white strigose base and *E.lasium* differs by the smaller basidiospores (8.9–14.5 × 5.1–8.7 μm, Largent et al. 2011). In addition, the two species are distant from *E.griseocarpum* following phylogenetic analysis. *E.puertoricense* Blanco-Dios (as *P.caribaea* T.J. Baroni & B. Ortiz in Baroni and Ortiz 2002) resembles *E.griseocarpum* by the brownish-grey coloured basidiomes but is separated by its broader basidiospores (12.5–16.5 × 8.3–11.3 μm, Q = 1.26–1.65, Baroni and Ortiz 2002). Moreover, *E.puertoricense* was discovered in a tropical habitat in Puerto Rico (Baroni and Ortiz 2002). The similar *E.japonicum* (Hongo) Hongo, described from Japan, is also reminiscent of *E.griseocarpum* in the brownish pileus but is distinguished by the much larger basidiospores (15–18.5 × 9–10.5 μm, Hongo 1959). The well-known *E.dysthales* (Peck) Sacc. also differs by the larger basidiospores (14–20 × 7.5–10 µm, Mazzer 1976). *E.fulvolanatum* (Berk. & Broome) Blanco-Dios from Sri Lanka is not only separated by its type locality but also by the narrower basidiospores measuring 12–16 × 7–8 μm (Mazzer 1976). Two species in subgen. Pouzarella, recently described from geographically neighbouring India, viz. *E.peechiense* K. N. A. Raj & Manim. and *E.silvanum* K. N. A. Raj & Manim., have somewhat similar basidiomes as compared to *E.griseocarpum*; however, their ITS and LSU sequences are distinctly different (Raj and Manimohan 2017). The third Indian species *E.lomapadum* Manim., Joseph & Leelav. is readily recognised by the much smaller basidiospores measuring 11–13 × 6–9 μm (Manimohan et al. 1995). There were four other species in subgen. Pouzarella which showed some similarities to *E.griseocarpum*. *E.fibrillosipes* (Murrill) Noordel. & Co-David is distinguished by the much larger basidiospores (17–22 × 7.5–10 μm, Mazzer 1976). *E.subdeceptivum* Courtec. and *E.rotula* (Romagn.) Noordel. & Co-David are lignicolous (Mazzer 1976). *E.homomorphum* (Romagn.) Singer differs by the larger basidiospores (15–19 × 9–11.5 μm, Mazzer 1976).

**Figure 3.**
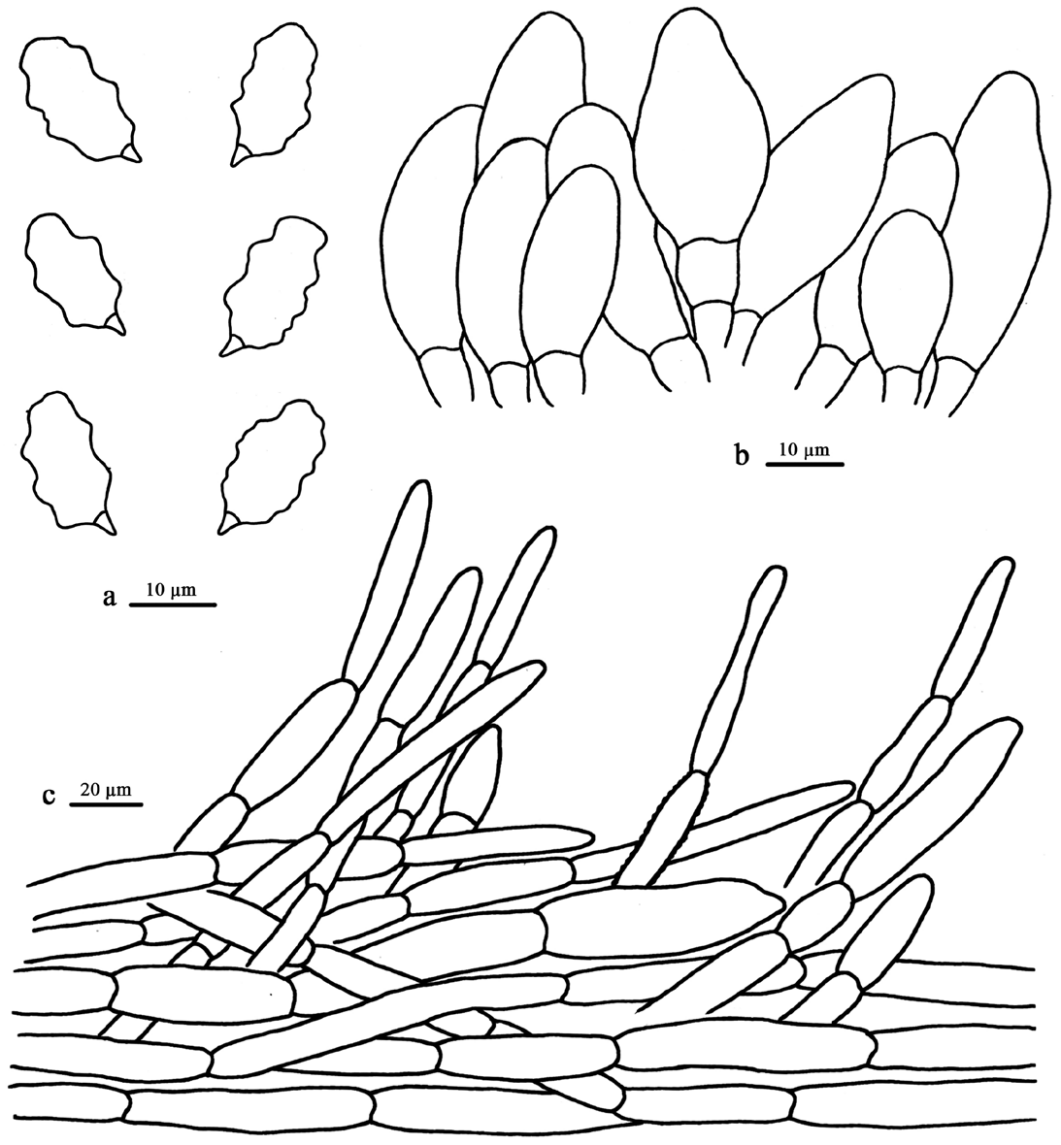
Microscopic structures of *E.griseocarpum* (drawn from the holotype). **a** Basidiospores **b** Cheilocystidia **c** Pileipellis.

### Entoloma rubropilosum

Taxon classificationFungiAgaricalesEntolomataceae

Xiao L. He & E. Horak sp. nov.

828699

Figs 1e, f , 4

#### Diagnosis.

*E.rubropilosum* is distinct due to its reddish-brown coloured pileus and stipe, large basidiospores (13–17 × 7.5–9.5 µm), broadly clavate cheilocystidia, distinctive thick-walled setiform caulocystidia and terminal cells of the pileipellis hyphae.

#### Type.

CHINA: SICHUAN PROV.: Yajiang County, Gexigou National Nature Reserve, 29°33'N, 100°50'E, elevation ca. 2950 m, 24 July 2013, He X.L. (SAAS 406, holotype).

#### Etymology.

*Rubropilosum*, refers to the reddish coloured fibrils on the pileus.

#### Description.

Pileus 7–20 mm broad, conical-convex, truncate conical to broadly campanulate, dark reddish-brown (8D2–8D3) at first, becoming greyish-orange to pale beige brownish (5B2–5C2), dry, slightly hygrophanous, densely covered by reddish-brown erect or suberect squamules and fibrils; fibrils much denser at disc, margin not striate or very slightly striate only. Lamellae adnate to sinuate, ventricose, up to 2.5 mm wide, relatively thick, with two tiers of lamellulae, brownish-pink when mature, with concolorous and entire edges. Stipe 40–73 × 0.8–2 mm, central, cylindrical, hollow, densely covered with rust reddish hairs or fibrils, very dark brown strigose at base. Odour and taste not distinctive.

Basidiospores (12.5–) 13–17 × 7.5–9.5 µm (*x* = 15.2 ± 0.5 × 8.5 ± 0.3 µm), Q = 1.53–1.98 (***Q*** = 1.76 ± 0.02), heterodiametrical, with 6–8 facets in profile and face views, sometimes multi-angled to nodulose, pale brownish, thick-walled. Basidia (32–) 38–45 (–50) × 12–16 µm, clavate, 4-spored. Aborted basidia inconspicuous. Lamellar edges sterile. Cheilocystidia 25–50 × 12–18 µm, broadly clavate, with faintly pale brownish, intracellular pigment, slightly thick-walled. Pleurocystidia absent. Pileipellis a trichoderm composed of brown hyphae; terminal cells 23–110 × 6–18 µm (diameter was measured at the base), slender setiform, gradually tapering towards subacute apex, sometimes subfusoid to somewhat bullet-shaped, thick-walled, with intraparietal and intracellular brown pigment; subpellis composed of cylindrical, relatively thin-walled hyphae, encrusted with yellow-brown pigment. Stipitipellis composed of loosely entangled, rather slender hyphae; terminal cells 45–120 × 5–11 µm (diameter was measured at the base), distinctly setiform with obtuse or subacute apex, thick-walled, with intraparietal and intracellular brown pigment. Oleiferous hyphae absent. Clamp connections absent.

#### Habitat.

Scattered on soil amongst decaying litter in broadleaf forest dominated by *Quercus* or in mixed forest with *Quercus*, *Betula*, *Rhododendron* and *Abies*, also on soil in bamboo forest.

#### Additional collections examined.

CHINA. SICHUAN PROV.: Yajiang County, Gexigou National Nature Reserve, 29°33'N, 100°50'E, elevation ca. 2950 m, 24 July 2013, He X.L. (SAAS 765); 24 July 2013, He X.L. (SAAS 706); 3 August 2014, He X.L. (SAAS 1488, SAAS 1112, ZT 14179). TIBET: Linzhi, Lulang, 29°94'N, 94°79'E, elevation ca. 3800 m, 18 September 2014, He X.L. (SAAS 1618, SAAS 1087); Linzhi, Kadinggou, 29°50'N, 93°26'E, elevation ca. 2950 m, 24 September 2014, He X.L. (SAAS 1456).

#### Comments.

The setiform terminal cells of pileipellis and stipitipellis place *E.rubropilosum* in sect. Pouzarella. It is readily recognised in the field. A few species of Entolomasubgen.Pouzarella with reddish-brown fibrils or squamules have been reported in literature (Mazzer 1976; Baroni et al. 2008). *E.ferreri* (T.J. Baroni, Perd.-Sánch. & S.A. Cantrell) Noordel. & Co-David is distinguished by dark blackish stains on the pileus caused from handling and non-setiform pileocystidia and caulocystidia (Baroni et al. 2008). *E.strigosissimum* (Rea) Noordel. is separated by the larger basidiospores [15–19 (23) × 8.5–10.5 (11.5) µm, Mazzer 1976]. *E.squamifolium* (Murrill) Singer might be confused with *E.rubropilosum* due to the ferruginous hairs on the stipe (Mazzer 1976). However, *E.rubropilosum* can be distinguished by the setiform pileocystidia and caulocystidia. Furthermore, type of *E.squamifolium* was collected in a tropical location. The recently described *E.wayanadense* K. N. A. Raj & Manim. from India, also discovered in a tropical area, is similar to *E.rubropilosum* in its greyish-orange pileus with long hairs and the setiform terminal cells of pileipellis, but differs by the absence of cheilocystidia. In addition, the partial ITS sequence (419 bp, KY 643748) of *E.wayanadense* is quite different from that of *E.rubropilosum* (Raj and Manimohan 2017).

**Figure 4.**
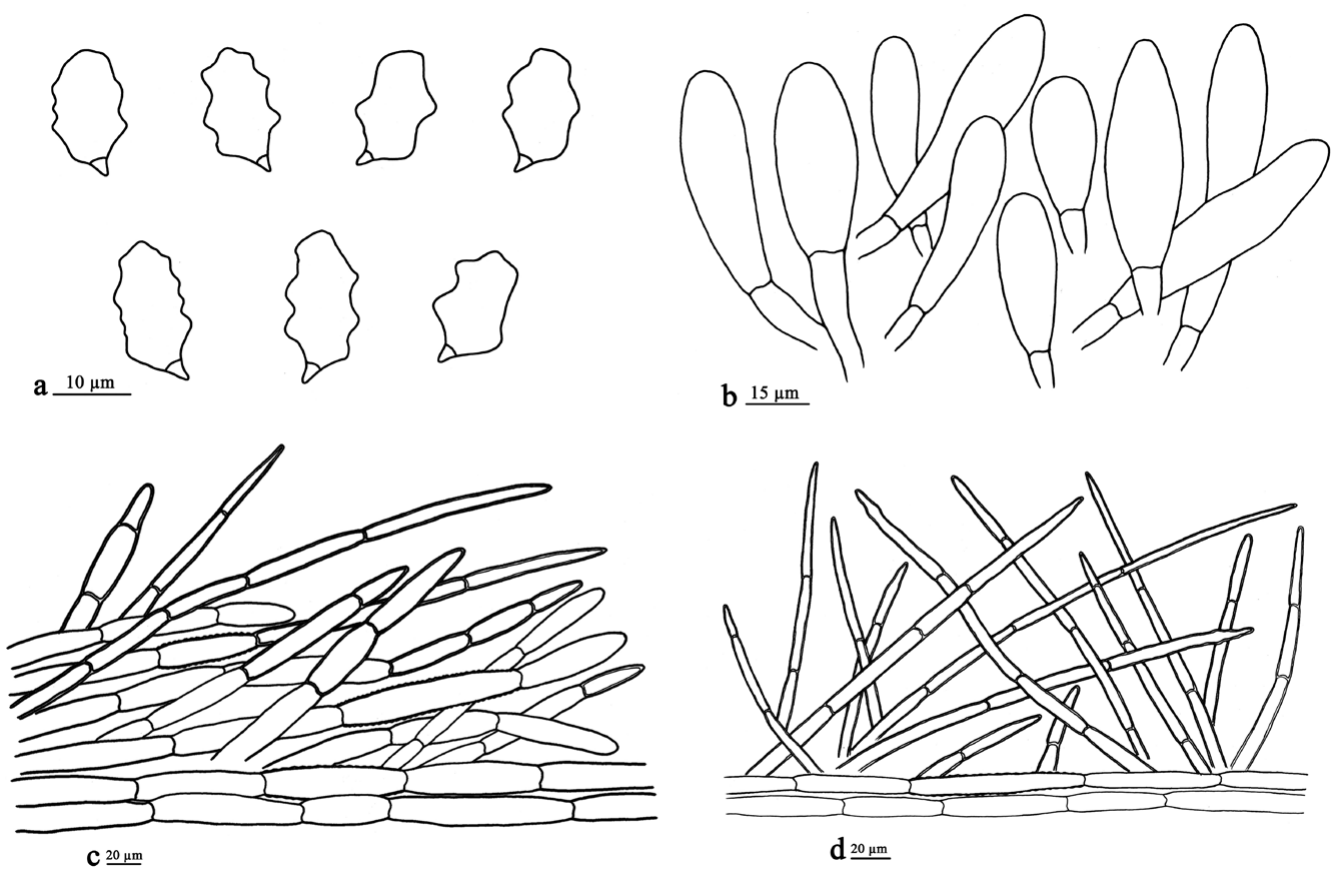
Microscopic structures of *E.rubropilosum* (drawn from the holotype). **a** Basidiospores **b** Cheilocystidia **c** Pileipellis **d** Stipitipellis.

### Key to the species of Entolomasubgen.Pouzarella described from China

**Table:** 

1	Pileus reddish-brown or greenish-brown	**2**
–	Pileus greyish-brown	**3**
2	Pileus covered with reddish-brown suberect fibrils	*** E. rubropilosum ***
–	Pileus greenish-brown with reddish tinge, zonate	*** E. changchunense ***
3	Pileus covered with appressed or suberect silvery fibrils	**4**
–	Pileus squamulose or covered with suberect brownish fibrils	**5**
4	Pileus covered with appressed silvery fibrils	*** E. subaraneosum ***
–	Pileus covered with suberect silvery fibrils	**6**
5	Pileus fibrillo-squamulose or squamulose-tomentose, growing in tropical forest	*** E. crassicystidiatum ***
–	Pileus covered with erect or suberect fibrils	**7**
6	Pileus pale brownish with pinkish tinge, basidiospores larger, average (16.8 ± 0.5) × (10.8 ± 0.3) µm	*** E. tenuissimum ***
–	Pileus greyish-brown, basidiospores smaller, average (15.5 ± 0.5) × (8.8 ± 0.3) µm	*** E. erectoides ***
7	Average spore length less than 13 µm	*** E. furfuraceum ***
–	Average spore length more than 13 µm	**8**
8	Pileus greyish-brown, striate from entire margin to near centre	*** E. griseocarpum ***
–	Pileus peach brown, not striate	*** E. yunnanense ***

## Molecular analysis

**Figure 5.**
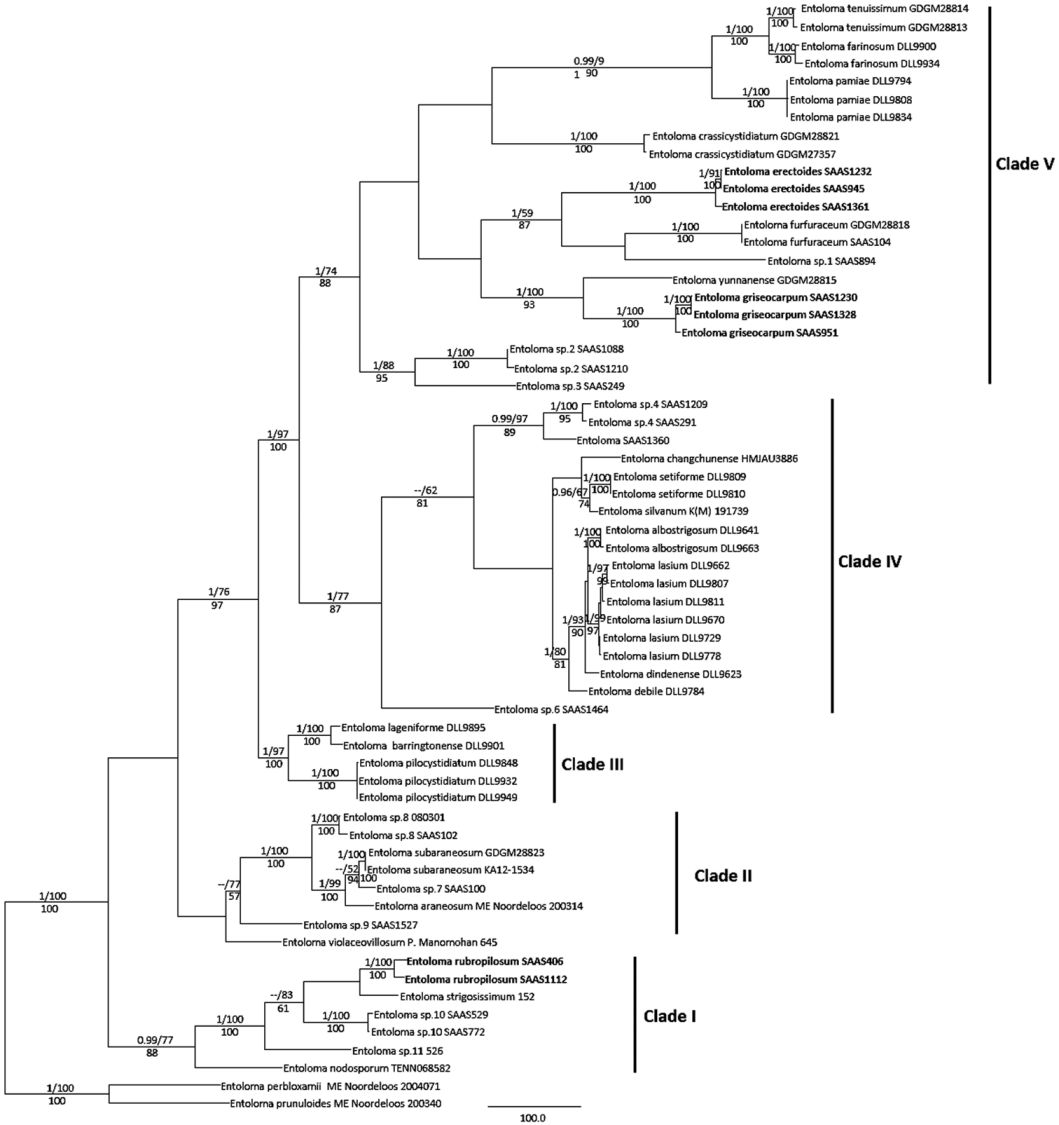
Phylogenetic relationships of Entolomasubgen.Pouzarella species inferred from the combined ITS, LSU, mtSSU and RPB2 dataset (new species are in bold). Bayesian posterior probability values (BPP > 0.90) and MP BS support values (> 50%) are indicated above branches as BPP/BS; RAxML BS support values (> 50%) are listed below branches.

A total 76 sequences were generated in this study and they were deposited in GenBank. The combined dataset in the molecular analyses is composed of 62 specimens and 2925 aligned sites. MP, ML and Bayesian analyses produced almost the same topologies except for the unsupported branches and the MP tree is shown (Fig. 5).

For the ITS sequences used in the analyses, both the size of the entire ITS1-5.8-ITS2 region as well as for ITS1 and ITS2 were separately compared. It is remarkable that the sizes of the entire ITS region and ITS1 were significantly divergent. In general, the total length of ITS sequences in subgen. Pouzarella ranged from 591 bp to 1086 bp. ITS1 was highly variable in length, while the length of ITS2 is relatively conserved within subgen. Pouzarella. ITS1 and ITS2 spacer varied from 229 to 690 bp and from 202 to 255 bp, respectively. However, it is noteworthy that two groups of the whole ITS region and ITS1 spacer could be partitioned in length. One group was varying from 591 bp to 709 bp and the other from 967 bp to 1086 bp. For the 5.8S region, 159–162 bp were yielded. Despite 5.8S is highly conserved, 4 indels and 11 nucleotide substitutes were found in this region. Regarding RPB2 sequences, their length was considerably conserved.

The three new species in this study were placed in different clades, showing they are quite different from each other. *E.rubropilosum* in relatively close to *E.strigosissimum* in the analyses, but similarity of their ITS sequences is only 84%. *E.griseocarpum* is grouped with *E.yunnanense* J.Z. Ying, but more than 150 different bases were observed in their ITS sequences. *E.erectoides* and *E.furfuraceum* nested in the same clade; however, more than 100 different bases were detected amongst their ITS sequences.

## Results and discussion

In the present study, three new species of Entolomasubgen.Pouzarella viz. *E.griseocarpum*, *E.erectoides* and *E.rubropilosum*, are reported from southwest China. The description is based on morphological and molecular characters. Together with the seven afore-mentioned species, ten taxa of Entolomasubgen.Pouzarella are now recorded for China. Five of those have been discovered in the northeast of China, four in southwest China and only one was reported from southern China (Ying 1995; He et al. 2013).

In the phylogenetic analyses, 35 taxa in subgen. Pouzarella were included and twenty phylogenetic species from China were recovered, suggesting a high species diversity in this geographical region. Five distinct clades (Clades I–V) were observed and the three new species are phylogenetically separated from each other. Based on morphological characters, *Pouzarella* (as a genus or subgenus of *Entoloma* s.l.) was divided into three sections (*Dysthales*, *Pouzarella* and *Versatiles*, Mazzer 1976; Noordeloos 1992). In our phylogenetic tree, Clade I corresponds to sect. Pouzarella while the taxa belonging to Clade II are accommodated in sect. Versatile. Except for several species of uncertain position, most members of Clade III, Clade IV and Clade V belong to the traditional sect. Dysthales morphologically. *E.rubropilosum* is nested in Clade I, which also includes *E.nodosporum* (G.F. Atk.) Noordel.and *E.strigosissimum*, as well as two still unknown species (*Entoloma* sp. 10 and *Entoloma* sp. 11) collected in China that morphologically fit the concept of sect. Pouzarella. These four species possess setiform pileocystidia and caulocystidia, somewhat reddish-brown or reddish fibrils on stipe and pileus. *E.erectoides* and *E.griseocarpum* are placed in Clade V.

The length of the ITS sequence of the species nested in Clade I, Clade II and Clade IV is relatively short, ranging from 591 bp to 709 bp. Available ITS sequences of Entolomasubgen.Pouzarella species in Clade V are recorded from 967 bp to 1086 bp. Unfortunately, no ITS sequences are available for comparison of the taxa belonging to Clade III. Eventual combination, referring both to morphological and molecular evidence, may in the future fundamentally change the classification of Entolomasubgen.Pouzarella.

## Supplementary Material

XML Treatment for Entoloma erectoides

XML Treatment for Entoloma griseocarpum

XML Treatment for Entoloma rubropilosum
